# Chitosan-Montmorillonite Polymer Composites: Formulation and Evaluation of Sustained Release Tablets of Aceclofenac

**DOI:** 10.3390/scipharm84040603

**Published:** 2015-10-22

**Authors:** Garima Thakur, Amrinder Singh, Inderbir Singh

**Affiliations:** Department of Pharmaceutics, Chitkara College of Pharmacy, Chitkara University, Patiala-Chandigarh Highway, Rajpura-140401, Patiala, Punjab, India; garimathakur89@yahoo.co.in (G.T.); amrinder3066@gmail.com (A.S.)

**Keywords:** chitosan, montmorillonite, chitosan-montmorillonite polymer composites, sustained release, tablets

## Abstract

The present study reports the preparation and evaluation of polymer composites of chitosan and montmorillonite. The prepared polymer composites were evaluated for various powder properties and characterized by FTIR-ATR (Fourier Transform Infrared Spectroscopy- Attenuated Total Reflectance), XRD (X Ray Diffraction), and SEM (Scaning Electron Microscopy) techniques. Heckel and Kawakita equations indicated good compression characteristics of the composites. The polymer composites were employed in formulating sustained release tablets of aceclofenac. The formation of intercalated lamellar structures due to the entrapment of clay particles in the polymeric matrix network was found to be responsible for the drug release retardant behavior of the composites. The in vitro drug release data were fitted to various models like zero-order, first-order, Higuchi, Korsmeyer-Peppas, and Hixon and Crowell for studying the mechanism of drug release from the formulation. The value of release exponent (*n*) was found to range between 0.59 and 0.82, indicating non-Fickian (anomalous) drug release behavior. Swelling-induced diffusion of the drug through the polymer matrix and polymer matrix chain relaxation appeared to play a role in the release of the drug from the polymer composites.

## 1. Introduction

In the past decade, biodegradable and biocompatible polymers have created significant interest from both ecological and biomedical perspectives. The use of natural polymers and their semi-synthetic derivatives in drug delivery systems is an area of active research. Natural polymers remain the prime choice because they are inexpensive, readily available, capable of a multitude of chemical modifications, and potentially degradable and biocompatible [1,2]. One such biopolymer is chitosan which is a biocompatible, biodegradable, nontoxic, linear cationic copolymer composed of N-acetylglucosamine and d-glucosamine units. Chitosan is derived from the N-deacetylation of chitin and has a number of exposed amino groups which makes it a polycationic polysaccharide. Depending upon the extent of deacetylation, different grades of chitosan are available. Due to its gel-forming property, it has been used in the development of various drug delivery systems [3]. Chitosan has been extensively investigated for several decades for molecular separation, food packaging film, artificial skin, bone substitutes, water engineering, and so on, owing to its good mechanical properties, biocompatibility, biodegradability, multiple functional groups as well as solubility in aqueous medium. However, its properties such as thermal stability, hardness, and gas barrier properties are not good enough to meet those wide ranges of applications. For this reason, the chemical structure of chitosan containing multiple functional groups (hydroxyl, carbonyl, carboxyl, amine, amide) creates the possibility for new bonding between the chitosan chain and nano-filler particles like clay (bentonite, montmorillonite), silica, and carbon nanotubes [4].

Montmorillonite is aluminosilicate clay composed of tetrahedral layers of silica stacked between octahedral layers of alumina. The isomorphous substitution of Al^3+^ for Si^4+^ in the tetrahedral layer and Mg^2+^ for Al^3+^ in the octahedral layer results in a net negative surface charge on the clay. Montmorillonite has a large specific surface area and exhibits good adsorption, cation exchange, and drug carrying capabilities [5].

Biopolymer/clay composites have drawn attention owing to the enhancement of physical and/or chemical properties relative to the pure polymer. The synergistic effect of the biopolymer and clay as well as the strong interfacial interactions between them (e.g., electrostatic and hydrogen bonding interaction) could improve the mechanical, swelling, water uptake, thermal, drug-loading efficiency, and controlled release behavior of the pristine biopolymer matrices [6]. 

Aceclofenac is a non-steroidal anti-inflammatory drug (NSAID), which is commonly prescribed for the treatment of pain, rheumatoid arthritis, osteoarthritis, and ankylosing spondylitis. It is a weak acid with a pKa value of 4.7. It is practically insoluble in water and acidic environments. The oral absorption of aceclofenac is uniform, rapid, and complete with a bioavailability of nearly 100% and an elimination half-life of 2–4 h. It is reported to have a short biological half-life of 3.4 ± 0.7 requiring to be administered in 100 mg twice daily [7]. 

The Heckel analysis is a popular method for determining the volume reduction mechanism under the compression pressure. Powder packing with increasing compression load can be attributed to the particle rearrangement, elastic and plastic deformation, and fragmentation of the particle. Heckel analysis follows first-order reaction kinetics in relation to the densification of the powder bed. In the Kawakita equation, the particle density is not involved. The Kawakita equation is used to evaluate powder compression behavior using the degree of volume reduction [8].

The present study was designed to prepare chitosan (CS) and montmorillonite (MMT) polymer composites. The prepared polymer composites were evaluated for various powder properties viz. angle of repose, bulk density, tapped density, Carr’s compressibility index, Hausner’s ratio, swelling index, pH, loss on drying, and effective pore radius. Characterization of polymer composites was performed by FTIR-ATR (Fourier Transform Infrared Spectroscopy- Attenuated Total Reflectance), XRD (X Ray Diffraction), and SEM (Scaning Electron Microscopy) techniques. Heckel and Kawakita equations were employed for studying compression characteristics of the polymer composites. The polymer composites were employed to formulate sustained release tablets of aceclofenac. Formulated tablets were evaluated for various tablet parametric tests like diameter, thickness, hardness, friability, tensile strength, drug content, and in vitro dissolution studies. The formulated tablets were subjected to stability studies.

## 2. Materials and Methods

### 2.1. Materials

Medium molecular weight chitosan (CS) (MW = 92,700 g/mol) and montmorillonite K10 (MMT) were procured from Sigma-Aldrich, St. Louis, MO, USA. Aceclofenac was gifted by IPZA Pharmaceuticals, Patiala, Punjab, India. All other ingredients used were of analytical grade and were used as received.

### 2.2. Preparation of Polymer Composites 

The CS/MMT polymer composites were prepared as per the method prescribed by Wang et al. with some modifications [2]. Chitosan solution was prepared by dissolving chitosan in 2% (v/v) aqueous acetic acid. MMT was first allowed to hydrate in 50 mL distilled water and then added to 50 mL chitosan solution followed by stirring at 60 °C for 6 h. Three ratios of the CS/MMT composite were prepared i.e., 1:1, 4:1, and 1:4 labeled as CS/MMT 11, CS/MMT 41, and CS/MMT 14, respectively. The prepared CS/MMT composites were dried in hot air oven at a temperature not more than 60 °C for 48 h. The dried composites were stored in a desiccator till further use.

### 2.3. Powder Characterization

The prepared CS/MMT composites were evaluated for various powder characterization studies like a micrometric study (angle of repose, bulk density, tapped density, Carr’s compressibility index, Hausner’s ratio), swelling index, pH, loss on drying, and effective pore radius, and chemical characterization by FTIR-ATR, XRD, and DSC (Differential Scaning Calorimetry) studies and SEM analysis. The compression analysis study was conducted employing Heckel and Kawakita models. 

#### 2.3.1. Micrometric Studies

Bulk density and tapped density were calculated in accordance with the method described in Section 2.9.34 of the European Pharmacopoeia [9].

Carr index: It was computed from tapped and bulk density using the following equation:

Carr’s index = (Tapped Density − Bulk Density/Tapped Density) × 100

Hausner ratio: This was calculated from tapped and bulk density using the following expression:

Hausner ratio = Tapped Density/Bulk Density

#### 2.3.2. Swelling Index

Initial bulk volume of the composite powder was evaluated using a 100 mL graduated cylinder. Water was added in a sufficient quantity to produce uniform dispersion. The sediment volume of the swollen mass was measured after 24 h. The swelling index was calculated as

Swelling index = [(V_2_− V_1_)/V_1_] × 100

where, V_1_ and V_2_ are the volume of powder before and after hydration, respectively.

#### 2.3.3. pH

A 1% dispersion (w/v) of the polymer composites of different ratios was prepared in distilled water and the pH was determined individually using a digital pH meter at 37 ± 2 °C.

#### 2.3.4. Loss on Drying 

Initial weight of polymer composite sample was weighed (W_1_) followed by heating in a convection oven (Microsil, Ambala, India) at 100 ± 5 °C until a constant weight was obtained. Sample was cooled in a dessicator and reweighed (W_2_). % Loss on drying (%LOD) was calculated by

%LOD = [(W_1_ − W_2_)/W_1_] × 100

#### 2.3.5. Effective Pore Radius 

Effective pore radius (R_eff.p_) was determined using the method reported by Goel et al. [10]. A micropipette tip (2 mL, transparent) was completely filled with powder and weighed (W_i_). Then n-hexane (with the surface tension (γ) of 18.4 mN/m) was poured drop wise on bed top till the solvent filtered out at the bottom of the tip. The tip was reweighed (W_f_) and effective pore radius was calculated by

R_eff.P_ = (W_f_ − W_i_)/2πγ

### 2.4. Fourier Transform Infra Red- Attenuated Total Reflectance

The FTIR-ATR spectra of samples were obtained using an FTIR-ATR spectrophotometer (Alpha, Bruker, Japan) by KBr pellet method. The samples were scanned in the spectral region of 4000 cm^−1^ to 400 cm^−1^.

### 2.5. X-Ray Diffraction 

The XRD studies were performed using glass tubing with a Cu anode and graphite monochromator and the diffractometer (X-Pert Pro, PANalytical, Almelo, Netherlands) was operated at 40 mA and 40 kV. All the samples were placed randomly on a glass slide. The signals of the reflection angle of 2θ were recorded from 0° to 60° at a scanning rate 0.21°/sec.

### 2.6. Scanning Electron Microscopy 

The scanning electron micrographs of the surface morphology of polymer composites were accumulated using SEM (S 4300 Hitachi, Illinois, USA). The samples were stuck on a specimen holder using a silver plate, and then coated with palladium in a vacuum evaporator. An accelerator potential of 10 kV was used.

### 2.7. Compression Studies

Compression characteristics of the prepared polymer composites were studied by Heckel and Kawakita equations [11]. Pellets (200 mg) were prepared by compressing the prepared conjugates for 30 s with predetermined loads on a hydraulic press (CAP15T-1233, PCI Analytics, Mumbai, India). Their masses (m) and dimensions were then determined respectively, and their relative densities (R) were calculated using the equation:

R = m/(V_t_ × ρ_s_)

where, V_t_ is the volume (cm^3^) of the tablet and ρ_s_ is the particle density (g/cm^3^) of the solid material.

#### 2.7.1. Heckel Function

The deformation mechanism of the polymer composites was determined using the Heckel model, as it is widely used for relating the relative density, D, of a powder bed during compression to the applied pressure, P. The Heckel equation is written as follows

ln [1/(1 − D)] = KP + A

where, K, the slope of the straight line of the plot represents the reciprocal of the mean yield pressure, and P, of the material. From the intercept A of the plot, the relative density, D_A_, can be from the following equation:

D_A_ = 1 − e^–A^

Relative density of the powder at the point when the applied pressure equals zero, D_0_, is used to describe the initial rearrangement phase of densification as a result of die filling.

D_B_ = D_A_− D_0_

Relative density, D_B_, describes the phase of rearrangement at low pressures and is the difference between D_A_ and D_0_.

#### 2.7.2. Kawakita Function

In the Kawakita equation, the particle density is not introduced in the calculations since the model operates on the relative change in volume which gives the same result whether the relative or the absolute volume is used. The Kawakita equation is used to study powder compression using the degree of volume reduction (C) and is written as

C = (V_o_− V_p_)/V_o_= abP/(1 + bP)

The equation can be rearranged as

P/C = P/a + 1/ab

where, V_o_ is the initial bulk volume of the powder and V_p_ is the bulk volume after compression. Constant ‘a’ is equal to the minimum porosity of the material before compression while constant ‘b’ is related to the plasticity of the material. The reciprocal of ‘b’ gives the pressure term P_k_*,* which is the pressure required to reduce the powder bed by 50%.

### 2.8. Formulation of Sustained Release Tablets

Sustained release tablets of aceclofenac were formulated by the direct compression method. The prepared CS/MMT polymer composites were used as release retardant in the tablet formulation (Table 1). The specified quantity of the drug, diluent, and CS/MMT composite were weighed accurately, passed through 60 mesh sieves (250 µm opening size), and mixed by tumbling in a polybag for 15 to 20 min. The blend was lubricated with talc and magnesium stearate, mixing was done for an additional 5 min, and the resulting powder mixture was compressed into tablets using a multi-punch tabletting machine (AK Industries, Nakodar, Punjab, India) using 8.5 mm biconcave round set of die and punches. 

### 2.9. Evaluation of Sustained Release Tablets

The prepared tablets were evaluated for various tablet parametric tests, viz. diameter, thickness, hardness, friability, tensile strength, drug content, in vitro dissolution, and stability studies.

#### 2.9.1. Diameter and Thickness

A calibrated Vernier caliper (Mitutoyo, Kawasaki, Japan) was used to determine the diameter and thickness of tablets.

#### 2.9.2. Hardness

The tablet hardness is the force required to break a tablet in a diametric compression force. A Monsanto Hardness tester (EI Instruments, Haryana, India) was used in this study. This tester applies force to the tablet diametrically. The test was performed on six tablets and the average was calculated.

#### 2.9.3. Friability

The friability of 20 tablets was measured using a Roche Friabilator (Model 902, EI Instruments, Haryana, India). As per USP 30-NF 25, 20 tablets were weighed and rotated at 25 rpm for 4 min. Tablets were taken out, dusted, and reweighed. The weight loss was calculated and the percentage friability was calculated by the formula:

Percentage friability = [(W_I_ − W_F_)/W_I_] × 100

where, W_I_ is the combined initial weight and W_F_ is the combined final weight of the tablets.

#### 2.9.4. Tensile Strength

The tensile strength of tablets is the force required to break a tablet by compressing it in the radial direction. Tensile strength (T) is calculated using the equation:

T = 2F/πdt

where F is the crushing load, and d and t signify the diameter and thickness of the tablet.

#### 2.9.5. Drug Content 

Drug content uniformity was determined by pulverizing ten tablets. A quantity of powder equivalent to 100 mg aceclofenac was shaken with 100 mL of phosphate buffer saline (pH 6.8) for 30 min. The contents were filtered through a 0.45 µm membrane filter, diluted, and analyzed at 275 nm using a ultraviolet (UV) double beam visible spectrophotometer (2202, Systronics, Gujarat, India).

### 2.10. In Vitro Dissolution Studies

In vitro dissolution of the tablets was studied in the USP XXIV dissolution apparatus II (DS 8000, Lab India, Mumbai, India) employing a paddle stirrer at 50 rpm and 25 mm depth using 900 mL of phosphate buffer (pH 6.8) at 37 ± 0.5 °C as a dissolution medium. Aliquots of 5 mL each were withdrawn at specified time intervals and replaced with equal volume of fresh medium. The withdrawn aliquots were filtered through a 0.45 µm membrane filter and analyzed for drug content using a UV double beam visible spectrophotometer (2202, Systronics, Gujarat, India) at λ_max_ 275 nm. Drug concentration was calculated and expressed as cumulative percent of the drug released.

The in vitro drug dissolution data was fitted to various models such as zero-order (cumulative % drug release vs. time), first-order (log cumulative % drug remaining vs. time), Higuchi (cumulative % drug release vs. square root of time), Korsmeyer-Peppas (log cumulative % drug release vs. log time), and Hixon and Crowell (cube root of cumulative % drug remaining vs. time) models to ascertain the mechanism of drug release.

### 2.11. Stability Studies

The stability studies for the selected prepared batches were carried out in a stability chamber. During the full duration of the study, temperature and relative humidity of about 40 ± 2 °C and 75% relative humidity, respectively, were maintained. The effect of temperature and time on the physical characteristics of tablets was evaluated for assessing the stability of the prepared formulation. The formulations were analyzed at day 0, and 1, 2, and 3 month time intervals for hardness, friability, and drug content.

## 3. Results and Discussions

### 3.1. Micrometric Studies

The results of the micromeritic studies for CS/MMT polymer composites are listed in Table 2. The flow property of the CS in CS/MMT 14 was improved by the addition of MMT as compared to other formulations. CS/MMT 14 showed excellent flow properties as indicated by the values of angle of repose (27.21 ± 0.01) to the CS/MMT 14 and 11 i.e., (29.45 ± 0.04) and (31.14 ± 0.03), respectively. Carr’s index of CS/MMT 14 and 41 showed values of 9.61 ± 0.72 and 10.65 ± 0.58, respectively, denoting that these formulations were of excellent flowability as compared to CS/MMT 11 (12.90 ± 0.42) which also showed good flow properties. Hausner’s ratio for CS/MMT 14 and 41 showed that powders with low inter-particle friction had ratios of approximately (1.11 ± 0.15) and (1.12 ± 0.08), indicating excellent flow properties as compared to the CS/MMT 11 (1.15 ± 0.11) showing good flow properties. The swelling index of CS/MMT 41 and 11 was found to be 95% and 83%, respectively, which was better than the swelling index of CS/MMT 14, i.e., 78%. Effective pore radius was found to be 14.84 ± 0.95 µm, 12.8 ± 0.88 µm, and 11.3 ± 1.02 µm for CS/MMT 41, 11, and 14 respectively.

### 3.2. FTIR-ATR Analysis

**Figure 1:**
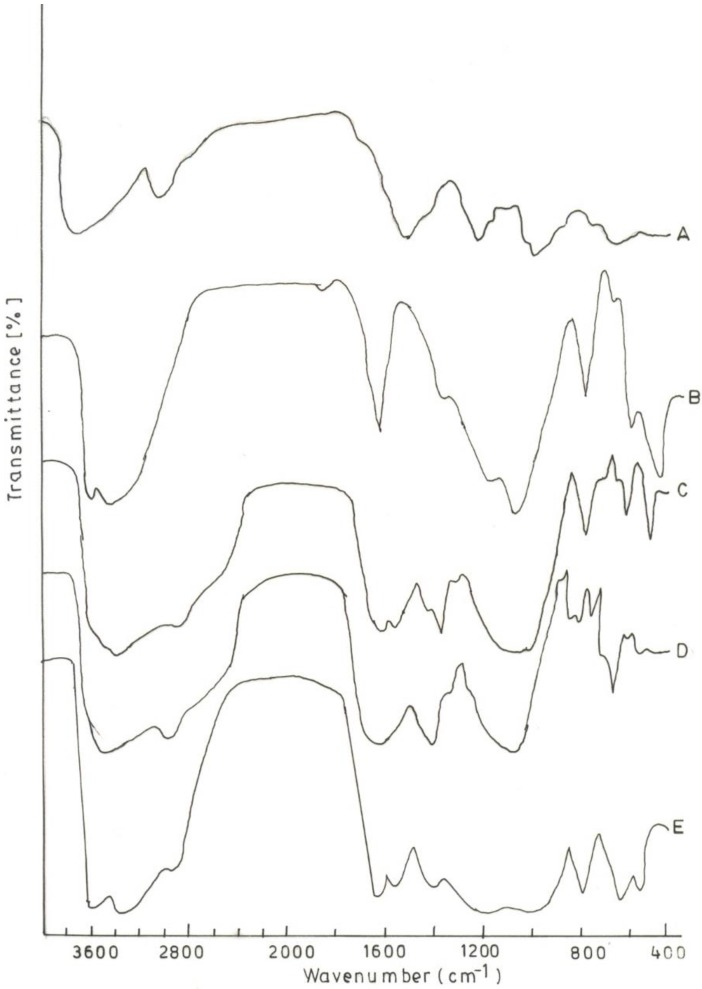
FTIR spectra of (**A**) Chitosan (CS); (**B**) Montmorillonite (MMT); (**C**) CS/MMT 11; (**D**) CS/MMT 41, and (**E**) CS/MMT 14.

The FTIR spectra of aceclofenac showed characteristic bands at 3319.3 cm^−1^ (N–H stretching), 2970.2, and 2935.5 cm^−1^ (O–H stretching), 1716.5 cm^−1^ (C–O stretching), 1589.2 cm^−1^ (skeleton vibration of aromatic C–C stretching), 1506.3 cm^−1^ (in plane bending for N–H), 1380 cm^−1^ (O–H in plane bending), 1280.6 cm^−1^ (C–N aromatic amine), 944 cm^−1^ (O–H out plane bending), and 746 cm^−1^ (out plane bending for N–H). The FTIR-ATR spectroscopy was used to analyze the interaction between CS and MMT in the polymer composites. Figure 1 shows that the peak at 3422 depicts the O-H stretching in the CS chain. The peak at 2914 cm^−1^ is due to the aliphatic C-H stretching in CS. The sharp peak at 1384 cm^−1^ shows the CH_3_ symmetrical deformation pattern [12]. The amide group is represented by the band at 1642 cm^−1^ and the amino group by 1258 cm^−1^. The peak at 1079 and 897 cm^−1^ confirms the saccharide structure of chitosan. The broad peak in MMT at 3449 cm^−1^ is due to interlayer and intralayer H-bonding upon stretching, while the O-H stretching is indicated by the peak at 3619 cm^−1^. The O-H bending of the H_2_O present in the MMT is shown by the peak at 1633 cm^−1^. The bands around 520–560 cm^−1^ are due to Si-O bending vibrations in MMT while the 1134 cm^−1^ band is due to the Si-O stretching vibrations. The formation of the intercalated structure in the polymer composite could be due to the interaction between the silicate hydroxylated edge group of MMT and the amino group of the CS chain [4]. At 1555 cm^−1^, the deformation vibration of the protonated amine group in the CS is shifted towards the lower frequency value indicating an electrostatic interaction between such groups and the negatively charged sites in the clay structure [13].

### 3.3. XRD Analysis

**Figure 2:**
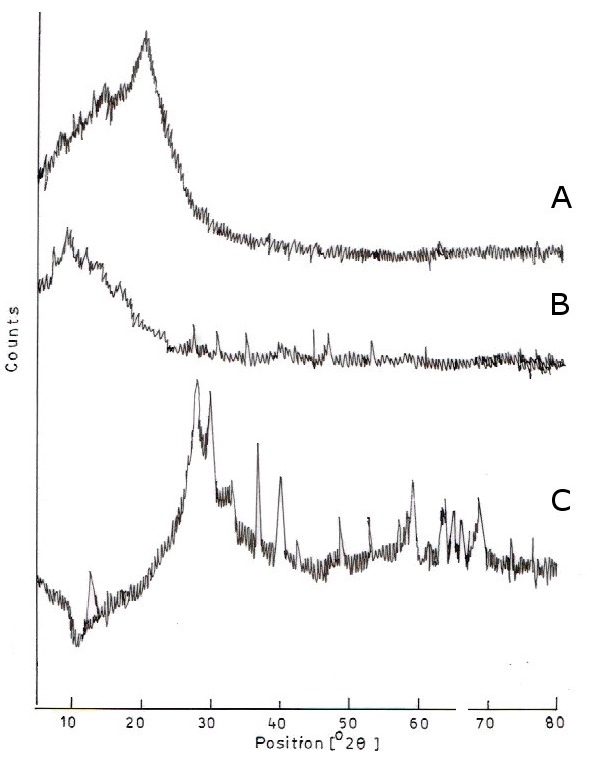
XRD pattern of (**A**) Chitosan; (**B**) Montmorillonite; and (**C**) CS/MMT polymer composite.

Figure 2 shows the evolution of XRD patterns as a function of the CS/MMT mixing ratio. CS shows a characteristic peak at 2θ = 20.95° while MMT shows a peak at 2θ = 8.54° and corresponds to a d-spacing of 4.3A°. The formation of the polymer composite can be seen in the chart at the peak 2θ = 7.4° and at a d-spacing of 10.13A°.

The reason for the shifting of the MMT peak at a lower angle clearly indicates the intercalation of MMT with CS. The increase of 4.3–10.13 A° in the interlayer spacing for the composite also indicates the intercalation of the bilayers of CS with interlayers of MMT and formation of an intercalated structure [14]. This explanation can also be supported by explaining that CS has one amino and two hydroxyl functional groups which interact with the silicate layers of MMT, which have hydroxylated edge groups. This interaction can form hydrogen bonds with the silicate. This strong interaction is believed to be the main driving force for the coagulation of MMT in the CS matrix to form a stacked structure [15]. Thus, it is observed that the crystalline structure of the polymer composite formed is greater than the crystallinity of the individual CS and MMT polymers.

### 3.4. SEM Analysis

**Figure 3:**
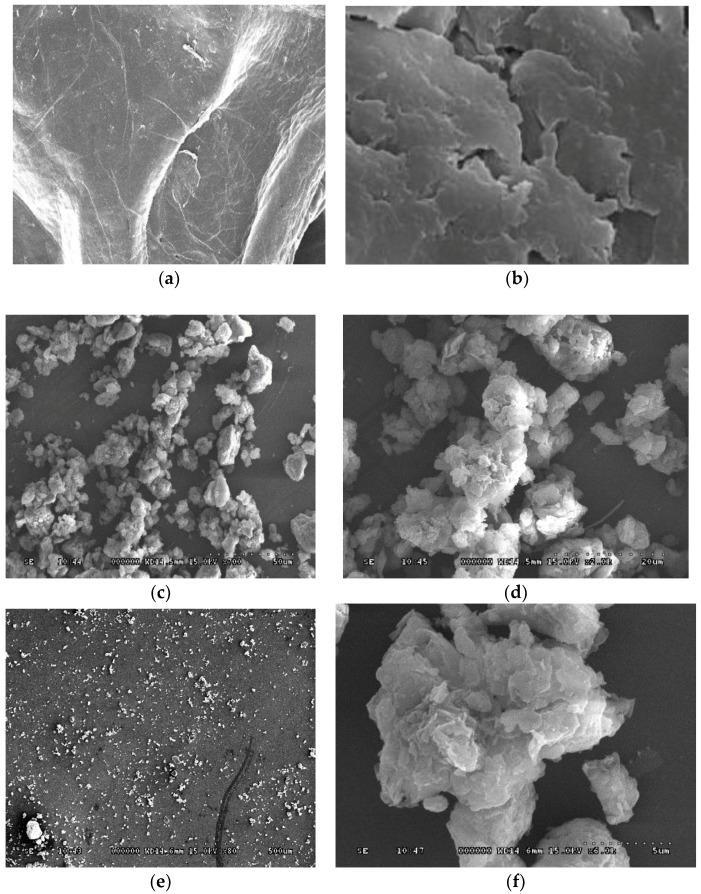
Scaning Electron Microscopy (SEM) images of (**A**) pure chitosan, (**B**) pure montmorillonite and (**C**–**F**) CS/MMT polymer composite at different magnifications.

The surface morphology of CS/MMT polymer composites studied by SEM analysis are shown in Figure 3. Pure CS has a smooth surface as shown in the SEM image. However, smoothness of the CS surface was found to decrease with the polymer matrix network. SEM images show the presence of CS-rendered intercalated lamellar structures due to the entrapment and/or interaction of clay particles within the polymer matrix. SEM analysis results are in line with the FTIR results, indicating the presence of intercalated structures in the CS/MMT composite due to the electrostatic interaction of the amino and silicate groups CS and MMT, respectively. Tan et al. indicated the presence of stacked flakes, depicting the interaction between chitosan and montmorillonite in the CS/MMT biocomposites [13]. Celis et al. reported the presence of lamellar structures in montmorillonite chitosan bionanocomposites [16].

### 3.5. Compression Study

Mathematical compression models are usually normalized by including either the particle density or the initial volume in the equation to make the parameters of different substances comparable. European Pharmacopeia defines the particle density as the density that includes a solid fraction and the volume of the intra-particulate pores. The particle density is dependent on the method of measurement and can be determined as either by a pycnometer or the mercury porosimeter. However, if the density is acting both as a scale and a form parameter, the question arises as to whether a change in the calculated parameters is caused by a change in compressibility or by a difference in the particle density entered in the calculation. The two well-known compression models, the Heckel and the Kawakita, were used to carry out the studies.

#### 3.5.1. Heckel Function Analysis

**Figure 4:**
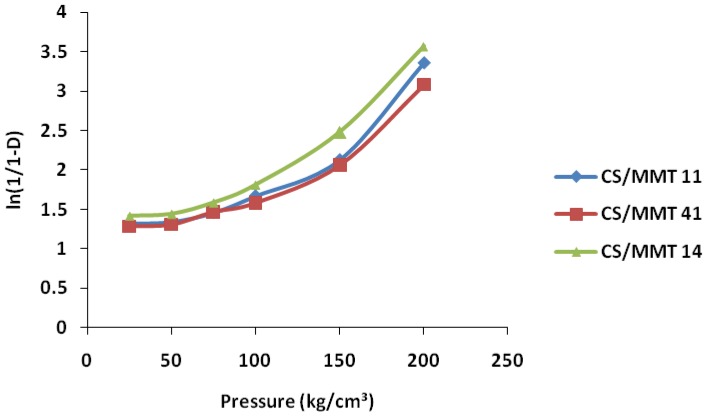
Heckel plots for the tablets incorporating CS/MMT polymer composites as a sustained release in different ratios.

Figure 4 shows the representative Heckel plots for the different ratios of the composites. The Heckel plots showed an initial linear portion with an increased slope at pressure of 100 MPa. The mean yield pressure values for the composites were calculated from the slope of the portion, showing the highest linearity of the Heckel plots, and the intercept, A, was determined from the extrapolation of the region. The values of D_A_ and D_B_ were calculated, respectively. The values of P_y_, D_o_, D_A_, and D_B_ for the formulations are presented in Table 3. The value of D_o_, which represents the degree of initial packing in the die as a result of die filling for the composites, indicates that the composites in the ratio 1:4, i.e., CS/MMT 14, exhibited the highest degree of packing in the die as a result of die filling, while the composites in the ratio 1:1, i.e., CS/MMT 11, exhibited the lowest values. The value of D_B_ represents the phase of rearrangement of the particles in the early stages of compression. D_B_ values tend to indicate the extent of fragmentation of particles or granules, although fragmentation can occur concurrently with plastic and elastic deformation of constituent particles. CS/MMT 14 and 41 exhibited higher values, while the one prepared in the ratio 1:1, i.e., CS/MMT 11, exhibited a lower value. This indicates that fragmentation occurs more with the composites containing a higher ratio of either MMT or CS. The values of D_A,_ which represent the total degree of packing achieved at zero and low pressures, were also in the rank order of CS/MMT 14 > CS/MMT 41 > CS/MMT 11 composites. This indicates that the composites in the ratio 1:4, i.e., CS/MMT 14, showed a higher degree of packing at low pressures. The mean yield pressure P_y_ is inversely related to the ability of the formulations to deform plastically under pressure. The result indicates that the composites in the ratio 1:1, i.e., CS/MMT 11, showed the fastest onset of plastic deformation while the composites in the ratio 1:4, i.e., CS/MMT 14, showed the slowest onset. Materials with high yield pressure are classified as brittle or fragmenting materials, whereas those with lower values are classified as plastically/elastically deforming materials. Generally during compression, plastic deformation and fragmentation are known to occur concurrently.

#### 3.5.2. Kawakita Function Analysis

**Figure 5:**
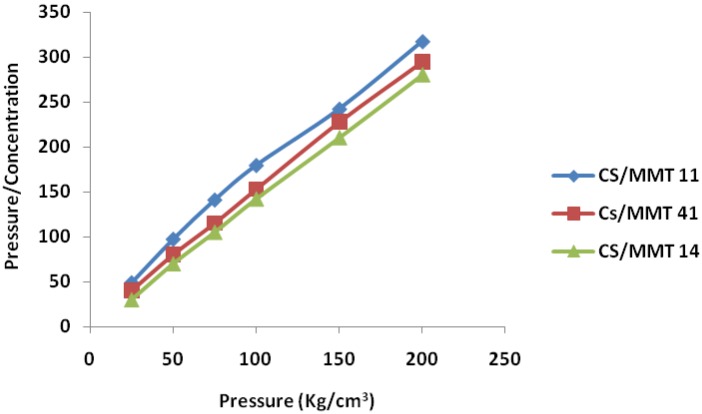
Kawakita plots for the tablets incorporating CS/MMT polymer composites as a sustained release in different ratios.

The Kawakita plots for the polymer composites are presented in Figure 5. A linear relationship was obtained at all compression pressures employed with a correlation coefficient of 0.999 for the composites prepared in different ratios. The values of *a* and *ab* were obtained from the slope and intercept, respectively. The value of (1-*a*) gives the initial relative density of the starch D_I_, while P_k_ values were obtained from the reciprocal of values of *b*. The values of D_I_ and P_k_ are shown in Table 4.

The value of D_I_ is a measure of the packed initial relative density of the formulation with the application of small pressure or tapping. The ranking of D_I_ for the composites was CS/MMT 11 > CS/MMT 41 > CS/MMT 14. The values of P_k_, which is an inverse measure of the amount of plastic deformation occurring during the compression process, for the composites were CS/MMT 11 > CS/MMT 41 > CS/MMT 14. Thus, the composites in the ratio 1:4 exhibited the highest amount of total plastic deformation, while composites in the ratio 4:1 exhibited the lowest values. The ranking was seen to be in the reverse order as that of the P_y_ values. It has been shown that while P_y_ relates to the onset of plastic deformation during compression; the P_k_ relates to the amount of plastic deformation that occurs during the compression process. Thus, the conjugates prepared in the ratio 1:4 showed the slowest onset of plastic deformation, but the highest total amount of plastic deformation.

### 3.6. Tablets Properties

Tablets require a certain amount of strength and resistance to friability to withstand the mechanical shock of handling during manufacturing, shipping, and packaging. The diameter and thickness of all the tablets varied from 8.48 to 8.54 mm and 3.79 to 3.86 mm, respectively. Hardness of the tablets formulated using the CS/MMT 14 as release retardant was found to vary from 4.5 to4 kg/cm^2^ compared to 3.5 to 4 kg/cm^2^ of tablets formulated using the CS/MMT 11 and 41. Percentage friability of all formulations was less than 1%, indicating good mechanical characteristics. Tensile strength was found to be increased in the tablets by incorporating a higher concentration of MMT, i.e., CS/MMT 14, as compared to CS/MMT 11 and 41 because MMT particles attach themselves between the chitosan chains, resulting in tighter packing and the granules are forced closer together during compression which will result in stronger packing of the granules and increase the tensile strength of the tablets. Table 4 shows the values of diameter, thickness, friability, hardness, tensile strength, and drug content of the sustained release tablets.

### 3.7. In Vitro Dissolution Studies

**Figure 6:**
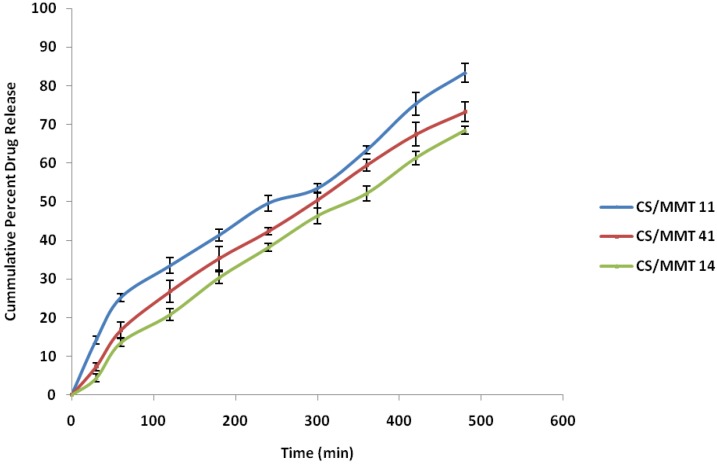
In vitro dissolution profile of the CS/MMT sustained release tablets.

The results obtained from the in vitro dissolution study are presented in Figure 6. It was observed that during the initial 60 min of the dissolution study, the polymer composite CS/MMT 11 showed 25.23% ± 1.02% drug release while the CS/MMT 41 and 14 polymer composites showed the release of drug as low as 16.76 ± 1.98 and 13.54% ± 0.75%, respectively. The drug release after 8 h of the dissolution study revealed that the polymer composite CS/MMT 14 showed the least drug release, i.e., 68.45% ± 2.34%, while the drug release profile of the polymer composites CS/MMT 41 and 11 showed a drug release of 73.23 ± 3.84 and 83.32% ± 4.37%, respectively. Results of the various tablet evaluation tests indicate that among the three samples used for the preparation of CS/MMT polymer composites, CS/MMT 14 and CS/MMT 41 are more effective ratios as compared to CS/MMT 11. CS/MMT 14 and 41 showed more sustained release effects. This might be explained by studying the electrostatic interaction between the cationic charges of CS and the anionic charges of MMT. This type of behavior may also be explained by saying that the CS polymer has a natural tendency to swell and the incorporation of MMT may accentuate the ability of the polymer composite to further increase the sustained release ability.

The regression coefficient (R^2^) values of the Higuchi plots for CS/MMT polymer composites 11, 41, and 14 were found to be 0.9719, 0.9914, and 0.9878, respectively, indicating the role of diffusion on the release of the drug. To further confirm the mechanism of drug release from the CS/MMT polymer composites, the in vitro dissolution data was subjected to the Korsmeyer-Peppas equation. The value of release exponent (*n*) was found to range between 0.59 and 0.82 indicating non-Fickian (anomalous) drug release behavior. Swelling-induced diffusion of the drug through the polymer matrix and the polymer matrix chain relaxation might be playing a role in the release of the drug from the CS/MMT polymer composites. Table 5 depicts the values of various release kinetics parameters for the sustained release tablets.

### 3.8. Stability Testing

The stability studies indicated that there was no significant change in tablet hardness, friability, and drug content as evident from Table 6.

## 4. Conclusions

Chitosan montmorillonite polymer composites were prepared and characterized by FTIR, XRD, and SEM studies. Formation of the intercalated structure in the polymer composites was attributed to the interaction of hydroxylated edge groups of montmorillonite with the amino groups of chitosan. Heckel and Kawakita analysis revealed the good compression characteristics of the polymer composites. Controlled release of the drug in the in vitro study revealed the matrix-forming ability of intercalated polymer composites to be responsible for retarding the drug release from the tablet formulation. The chitosan montmorillonite polymer composites could be used for the development of various drug delivery systems owing to their good compression and release retardant properties. It could also be explored for food and packaging applications.

## Figures and Tables

**Table 1:** Formulation codes for the formulation of sustained release tablets.

Ingredients (mg)	CS/MMT 11	CS/MMT 41	CS/MMT 14
Acelofenac	100	100	100
Lactose	70	70	70
CS/MMT polymer composite	25	25	25
PVP K 30	25	25	25
Talc	2.5	2.5	2.5
Magnesium stearate	2.5	2.5	2.5
**Total Weight (mg)**	**225**	**225**	**225**

CS (chitosan); MMT (montmorillonite).

**Table 2:** Values depicting the different micromeritic study parameters.

S. No.	Parameter	CS/MMT 11	CS/MMT 41	CS/MMT 14
1	Bulk density (g/cm^3^)	0.54 ± 0.09	0.42 ± 0.11	0.47 ± 0.12
2	Tapped density (g/cm^3^)	0.62 ± 0.05	0.47 ± 0.08	0.52 ± 0.10
3	Carr’s index (%)	12.90 ± 0.42	10.63 ± 0.58	9.61 ± 0.72
4	Hausner ratio	1.15 ± 0.11	1.12 ± 0.08	1.11 ± 0.15
5	Angle of repose (°)	29.45 ± 0.89	31.14 ± 0.07	27.21 ± 0.98
6	Swelling index (%)	83.3	95	78.57
7	pH	5.1	4.9	5.2
8	LOD (%)	12.5 ± 0.56	16.5 ± 1.02	4.5 ± 0.34
9	Effective pore radius (µm)	12.8 ± 0.88	14.84 ± 0.95	11.37 ± 1.02

LOD: Loss On Drying.

**Table 3:** Parameters derived from the Heckel and Kawakita plots for tablets incorporating CS/MMT polymer conjugates as a sustained release agent prepared in different ratios.

Samples	Heckel Analysis				Kawakita Analysis	
	D_0_	D_A_	D_B_	P_y_	D_I_	P_k_
CS/MMT 11	0.28	0.40	0.21	83.3	0.33	14.5
CS/MMT 41	0.29	0.52	0.23	90.9	0.30	5.6
CS/MMT 14	0.31	0.54	0.23	100	0.31	6.4

**Table 4:** Values depicting the size, friability, hardness, and tensile strength of the prepared sustained release tablets.

Formulation Code	Diameter (mm)	Thickness (mm)	Friability (%)	Hardness (kg/cm^2^)	Tensile Strength (mN/cm^2^)	Drug Content (%)
CS/MMT 11	6.73 ± 0.04	3.81 ± 0.04	0.71 ± 0.10	4.0 ± 0.11	0.60 ± 0.05	98.89 ± 0.82
CS/MMT 41	6.73 ± 0.04	3.75 ± 0.02	0.85 ± 0.12	4.5 ± 0.18	0.47 ± 0.09	99.12 ± 0.66
CS/MMT 14	6.74 ± 0.02	3.75 ± 0.04	0.91 ± 0.08	4.5 ± 0.27	0.73 ± 0.08	98.87 ± 0.91

**Table 5:** Release kinetics parameters of designed sustained release matrix tablets of aceclofenac.

Batch No.	Zero Order		First Order		Higuchi		Korsmeyer-Peppas			Hixon Crowell	
	R²	k_0_	R²	k_1_	R²	k_H_	R²	k_KP_	n	R²	k_HC_
**CS/MMT 11**	0.9694	0.1548	0.9348	−0.0004	0.9719	3.9467	0.9859	0.2938	0.5946	0.9652	−0.0038
**CS/MMT 41**	0.9662	0.1484	0.9871	−0.0012	0.9914	3.9577	0.9899	0.244	0.790	0.9953	−0.0033
**CS/MMT 14**	0.9629	0.1403	0.9865	−0.001	0.9878	3.8081	0.9787	0.6316	0.827	0.9946	0.003

**Table 6:** Stability testing data of the sustained release tablets.

Batch	Time Interval (months)	Test Parameters		
		Hardness (kg/cm^2^)	Friability (%)	Drug Content (%)
CS/MMT 11	0	3.56 ± 0.08	0.73 ± 0.05	99.1 ± 0.15
	1	3.51 ± 0.15	0.79 ± 0.09	98.7 ± 0.20
	2	3.47 ± 0.10	0.79 ± 0.07	98.1 ± 0.56
	3	3.41 ± 0.20	0.82 ± 0.03	97.8 ± 0.15
CS/MMT 41	0	3.80 ± 0.15	0.54 ± 0.05	98.92 ± 0.70
	1	3.71 ± 0.80	0.59 ± 0.06	98.67 ± 0.35
	2	3.65 ± 0.27	0.63 ± 0.02	98.1 ± 0.59
	3	3.50 ± 0.41	0.63 ± 0.07	97.34 ± 0.65
CS/MMT 14	0	4.25 ± 0.20	0.42 ± 0.02	98.99 ± 0.20
	1	4.11 ± 0.17	0.44 ± 0.04	98.61 ± 0.19
	2	4.04 ± 0.31	0.47 ± 0.09	98.28 ± 0.35
	3	3.98 ± 0.24	0.49 ± 0.01	97.91 ± 0.55
